# The impact of *Beauveria* species bioinocula on the soil microbial community structure in organic strawberry plantations

**DOI:** 10.3389/fmicb.2022.1073386

**Published:** 2023-01-11

**Authors:** Loredana Canfora, Małgorzata Tartanus, Andrea Manfredini, Cezary Tkaczuk, Anna Majchrowska-Safaryan, Eligio Malusà

**Affiliations:** ^1^Centre for Agriculture and Environment (CREA-AA), Council for Agricultural Research and Economics, Rome, Italy; ^2^Department of Plant Protection, National Institute of Horticultural Research, Skierniewice, Poland; ^3^Institute of Agriculture and Horticulture, Siedlce University of Natural Sciences and Humanities, Siedlce, Poland

**Keywords:** *Beauveria bassiana*, *Beauveria brongniartii*, soil bacteria community, soil fungal community, T-RFLP

## Abstract

**Introduction:**

The multifunctionality of microorganisms, including entomopathogenic fungi, represents a feature that could be exploited to support the development, marketing, and application of microbial-based products for plant protection. However, it is likely that this feature could affect the composition and dynamics of the resident soil microorganisms, possibly over a longer period. Therefore, the methodology utilized to evaluate such impact is critical for a reliable assessment. The present study was performed to evaluate the impact of strains of *Beauveria brongniartii* and *Beauveria bassiana* on soil bacterial and fungal communities using an approach based on the terminal restriction fragment polymorphism (T-RFLP) analysis.

**Materials and methods:**

Soil samples in the vicinity of the root system were collected during a 3-year period, before and after the bioinocula application, in two organic strawberry plantations. Specific primers were used for the amplification of the bacterial 16S rRNA gene and the fungal ITS region of the ribosome.

**Results and discussion:**

Data of the profile analysis from T-RFLP analysis were used to compare the operational taxonomic unit (OTU) occurrence and intensity in the inoculated soil with the uninoculated control. With regard to the impact on the bacterial community, both *Beauveria* species were not fully consistently affecting their composition across the seasons and fields tested. Nevertheless, some common patterns were pointed out in each field and, sometimes, also among them when considering the time elapsed from the bioinoculum application. The impact was even more inconsistent when analyzing the fungal community. It is thus concluded that the application of the bioinocula induced only a transient and limited effect on the soil microbial community, even though some changes in the structure dynamic and frequency of soil bacterial and fungal OTUs emerged.

## 1. Introduction

Biological control is an essential component of arthropod pest management in both organic and conventional cropping systems (Shah and Pell, 2003; Zehnder et al., 2007), and among the several studied organisms, entomopathogenic fungi (EPFs) are frequently considered for the control of soil pests (Butt, 2002). The evolution of policies worldwide aiming at the reduction of the use of synthetic pesticides has thus favored the development of plant protection products based on microorganisms, including EPF (de Faria and Wraight, 2007). However, the introduction of a bioinoculant to agricultural ecosystems can raise questions about its impact on non-target organisms. To address this, the European Union updated the guidelines to assess the risks of potential effects on non-target organisms during the registration process of biological pesticides (European Commission, 2013), being these organisms either closely related to the target species or being especially exposed, as it could be the case for soil native microbial populations.

On the other hand, the multifunctionality of microorganisms, including EPF-based bioinocula, represents an aspect that could further support the development, marketing, and application of microbial-based products (Harman, 2011; Kowalska et al., 2020). This has been recently shown particularly for EPF able to conduct also an endophytic development: acting as a plant-growth promoter (Raya–Díaz et al., 2017; Tall and Meyling, 2018) or supporting the control of soil-borne pathogens (Jaber, 2018). It is thus likely that all these functions could affect the composition and dynamics of the resident soil microorganisms, possibly over a long period. However, the methodology utilized to evaluate the impact of microbial inoculants on soil microbial community composition is a key to a reliable assessment (Canfora et al., 2014, 2016; Manfredini et al., 2021), due to the complexity of the soil environment (Nannipieri et al., 2017).

In the past few years, we have endeavored an effort to address the control of white grubs of *Melolontha* spp. in a region with a high incidence of this pest (Malusá et al., 2020). The application of EPF belonging to the species *Beauveria bassiana* (Balsamo -Crivelli) Vuill. (Ascomycota: Hypocreales) and *Beauveria brongniartii* (Saccardo) Petch is considered a suitable and effective method to achieve this goal (Zimmermann, 2007). Tartanus et al. (2021) reported the results of a study evaluating the behavior of these species in organic strawberry plantations in relation to the environmental conditions, their abundance after soil inoculation, and their impact on soil microbial communities carried out with a classical microbiological method. The present study was performed to evaluate the impact of the two *Beauveria* strains on soil bacterial and fungal communities using an approach based on terminal restriction fragment polymorphism (T-RFLP) analysis.

## 2. Materials and methods

### 2.1. Field trials

In total, two trials were carried out on strawberry plantations, managed according to the organic farming practices, located in the territory of Lubartów district (Lublin voivodeship, South-Eastern Poland), namely, at Brzostówka (51.4365°N, 22.7856°E) (following BRZ) and Nowa Wola (51.4177°N, 22.7238°E) (following NOW). Strawberry plants were planted in springtime (NOW, cv. Polka) or late summer (BRZ, cv. Senga Sengana) in 2014 in commercial fields for a typical 3-year crop. Both sites had a similar soil texture (sandy loam), classified as podsolic, but different pH values (5.3 and 6.8, for BRZ and NOW, respectively), salinity (0.7 and 1.0 dS m-1 for BRZ and NOW, respectively), and organic matter content (1.06 and 1.19% on dry soil weight for BRZ and NOW, respectively).

Both fields (about 1 ha) were highly infested by *Melolontha* spp. larvae, as determined by an initial assessment of their presence made by counting live grubs before planting the strawberries (on average, 2 larvae m^–2^ were found, 4-fold the acceptable damage threshold, i.e., 0.5 larvae m^–2^). The assessment was performed by collecting the soil samples from 25 cm × 25 cm × 30 cm (w:l:d) wells and checking for the presence of the grubs (minimum 8 holes from each repetition).

A randomized block design with four replicates (for a total of about 1,500 m^2^ per treatment) was established for the following treatments in both trials:

1) A *Beauveria bassiana* (Balsamo -Crivelli) Vuill. (Ascomycota: Hypocreales) strain (BB59, hereafter Ba) isolated from rhizospheric soil of an apple orchard located in Valle d’Aosta by the company CCS Aosta, (Aosta, Italy), which genomic sequence of ITS region of the ribosome has been deposited in the GenBank database and can be accessed to ID KT932307 (see below).
2) A *Beauveria brongniartii* (Saccardo) Petch strain (hereafter Br) isolated from the soil of a potato field highly infested by *Melolontha melolontha* in Romanów locality (Lublin voivodeship, Eastern Poland). The strain is deposited in the Fungal Collection of the Department of Horticulture and Plant Protection, Siedlce University of Natural Sciences and Humanities. The sequence of the ITS region of the ribosome has been deposited in the GenBank database and can be accessed to ID KT932309.

Control plots did not receive the bioinocula. The strains have not been tested before in the laboratory to assess their virulence in comparison with other strains. However, pot experiments carried out under controlled conditions confirmed that both were able to infect *M. melolontha* larvae (unpublished data). The *B. bassiana* bioinoculum was prepared by growing the fungus in a liquid medium based on the malt extract and glucose and formulated as a wettable powder into a carrier material made of a mixture of corn fibers and zeolite (1:10 w-w). *B. brongniartii* was grown on a solid substrate (barley kernels). The concentration of each of the two fungi in the inoculum was about 1⋅10^7^ spores⋅g^–1^. The colonized kernels were used as a carrier for application. *B. bassiana* was applied as an aqueous suspension, and to reduce the risk of damaging the fungal cells, a fan-less sprayer with large diameter nozzles was used to apply the equivalent of about 2,000 l⋅ha^–1^ of the bioinoculum water suspension. The applications were carried out near the plants’ rows. After each application, the soil was mixed on the surface with light hand hoeing.

Each treatment consisted of a dose of 45 kg⋅ha^–1^ applied to the soil. In the case of the trial NOW, the dose was split into four applications in the first year (starting at planting on 20 May 2014), with monthly intervals. For the trial BRZ, the dose was split into two applications with a 3-week interval in the first year (starting at planting on 30 July 2014). For both trials, a single application was performed for the following 2 years (mid-July and mid-May in 2015 and 2016, respectively).

### 2.2. Soil sampling and DNA extraction

Soil samples were collected as follows: mid-September 2014 (i.e., about 16 and 4 weeks after application for NOW and BRZ, respectively), mid-June and mid-September 2015 (i.e., before the second application and about 8 weeks after it), and mid-May and end of July 2016 (i.e., before the third application and about 8 weeks after it). The soil samples were collected from the vicinity of the plant’s root system, with an Egner’s sampler from a depth of 0–20 cm from about 25 points randomly distributed on each of the four plots for every treatment. These individual samples were merged to compose a laboratory sample (approximate weight of 1–1.5 kg).

DNA was extracted from 0.6 g of soil using the DNeasy PowerSoil^®^ DNA Isolation Kit (Qiagen Inc., Chatsworth, LA, USA) following the manufacturer’s instructions. DNA crude extract yields were calculated using Qubit^®^ 2.0 Fluorometer (Invitrogen, Thermo Fisher Scientific, Inc., Waltham, MA, USA), following the manufacturer’s instructions. DNA extraction was repeated in duplicates, and then, the DNA solutions were pooled. The extracted DNA was diluted to 10 ng μL^–1^ and stored at −20°C for the following analytical steps.

### 2.3. T-RFLP analysis of soil microbial community

Primers 63f and 1087r with a dye label VIC™ (Victoria, developed after the modifications of Aequorea victoria Green Fluorescent Protein) on the 5′ were used for the amplification of the bacterial 16S rRNA gene (Canfora et al., 2015). The operons ITS1 and ITS4, labeled with 6-FAM™ (6-carboxyfluorescein) dye, were used for the amplification of the fungal ITS region of the ribosome (primers sequence details are provided in Supplementary Table 1). PCRs were repeated in triplicate for each sample and were performed in a 30 μL volume with 50 ng of template DNA and 0.2 U of Taq Phusion hot start Taq DNA Polymerase (Platinum, Invitrogen, Thermo Fisher Scientific, Inc., Waltham, MA, USA). The PCR (for bacteria and fungi) was performed under the following conditions: 95°C for 5 min followed by 30 cycles of 95°C for 30 s, 55°C for 30 s, and 72°C for 1 min; the process was completed with a final extension step of 10 min at 72°C. The PCR products were separated on a 1.5% agarose.

The amplified products were purified with a Qiaquick PCR Purification Kit (Qiagen Inc., Chatsworth, LA, USA); after quantification with Qubit^®^ 2.0 Fluorometer (Invitrogen, Thermo Fisher Scientific, Inc., Waltham, MA, USA), 600 ng of amplified 16S rDNA was digested for 5 h with 20 U of *Taq*I and *Alu*I (Promega, Madisson, WI, USA) at 37°C and 65°C, respectively. For the ITS region of the fungal DNA, 600 ng of the amplified product was digested with 20 U of *Hin*fI and *Hae*III (Promega, Madisson, WI, USA) for 5 h at 37°C. A 50 ng aliquot of the digested products was resolved by the capillary electrophoresis on an ABI3500 Genetic Analyzer (Applied Biosystems, Thermo Fisher Scientific, Inc., Waltham, MA, USA) using LIZ600 as the size standard for GeneScan analysis. Fragment sizes from 55 to 500 bp were considered for profile analysis and determination of the operational taxonomic unit (OTU) numbers.

### 2.4. Data treatment and statistical analyses

Data obtained from T-RFLP analysis were used to compare the soil inoculated with *B. bassiana* and *B. brongniartii* with the uninoculated control. A derivative profile was created by the comparison of T-RFLP profiles from each restriction enzyme from duplicate DNA samples following the method reported in the study of Canfora et al. (2017). The quality of T-RFLP data was checked by GeneMarker software (SoftGenetics, LLC, State College, PA, USA). Each peak on the T-RFLP profiles is thought to correspond to a certain anonymous taxon referred to as an operational taxonomic unit (OTU), while the area of the peak is thought to correspond to the proportion of this OTU in the microbial community. Only fragments with fluorescence intensity of ≥55 arbitrary fluorescence units were considered. Alignment of the profiles was performed directly on the output table of the software GeneMarker, considering a range of ± 0.5 bp to discriminate peaks of consecutive sizes. The derivatives of terminal restriction fragment (T-RF) profiles of the different enzymes were combined and transformed into a binary vector, in which the intensity area of peaks was scored as strings, to be used for the analysis. The number of peaks was counted in each sample across the treatments. A heatmap was created on the retrieved number of peaks with a frequency of ≥3, using XLSTAT software 2020.2.3 (Addinsoft, 2020). The map included TRFs with at least one fragment present per sample, thus displaying the common fragments and highlighting the differences in terms of frequency (the presence or the absence of TRFs), as well as in terms of “intensities” (area of the peak). Considering that an error of ± 0.5 bp in discriminate peaks could occur when reading the bands, particularly comparing runs from different sampling periods, we assumed that peaks with ± 0.5 bp of difference correspond to the same OTU (i.e., 135.1 = 135.2 = 134.8; 139.1 = 139.2; 71.2 = 71.7).

## 3. Results

The T-RFLP data were analyzed based on the assumption that the T-RF number (i.e., each peak in the T-RFLP profiles) represents a different species/strain identified as an OTU. Indeed, while the Shannon H index can evaluate the OTU diversity, its identity remains unknown. Therefore, T-RFLP data were analyzed by assessing the presence/absence of each OTU (peak) and its intensity (as semiquantitative data) for both sites, displaying the occurrence of the common OTU (T-RF with at least one peak per treatment in each sample period) on heatmaps. To better appraise the stringency of the approach followed in the data elaboration, the total number of OTUs identified in the samples is also provided in the relevant tables with the number of OTUs utilized for the assessment.

### 3.1. Impact of inoculated species on soil bacterial community

**FIGURE 1:**
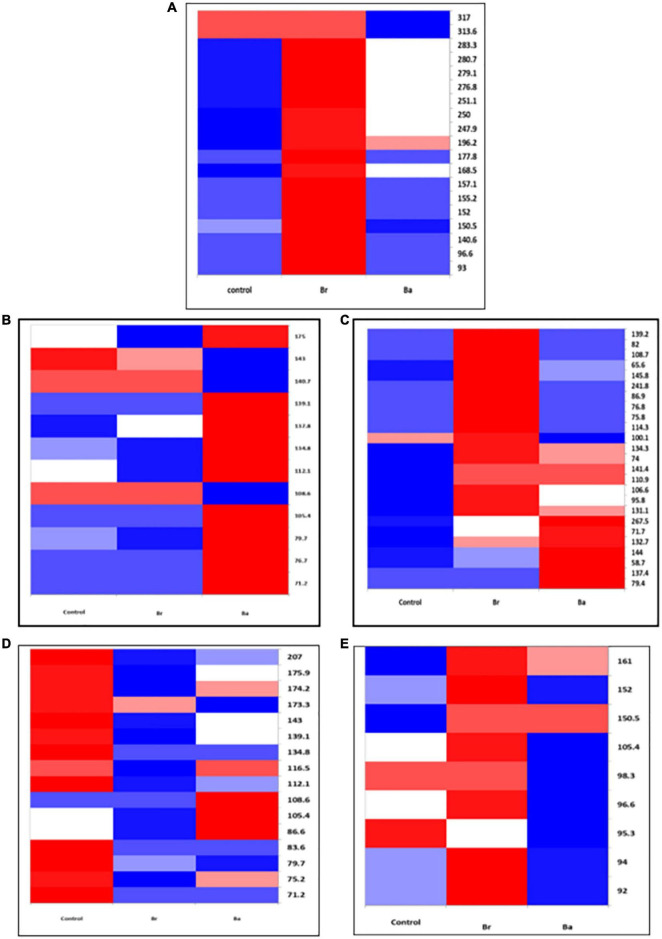
Heatmap displaying the occurrences of the common bacterial operational taxonomic units (OTUs) identified, as T-RFs, in the soils treated with *Beauveria brongniartii* (Br) and *Beauveria bassiana* (Ba) bioinocula and untreated (Control) in the field located at Nowa Wola (NOW) as sampled during the 3 years. The color represents the OTU intensity: Blue to red, min to max. The absence of OTU is displayed as white line. The intensity of the color represents the percentage of occurrence in the treatment. Samples were collected in **(A)** September 2014, **(B)** June 2015, **(C)** September 2015, **(D)** May 2016, and **(E)** July 2016.

**FIGURE 2:**
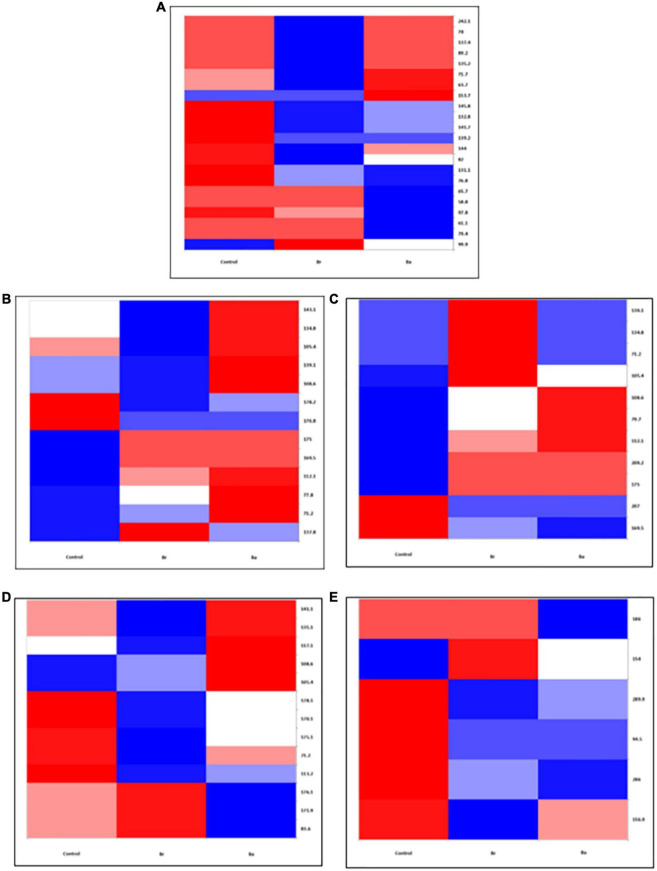
Heatmap displaying the occurrences of the common bacterial operational taxonomic units (OTUs) identified, as T-RFs, in the soils treated with *Beauveria brongniartii* (Br) and *Beauveria bassiana* (Ba) bioinocula and untreated (Control) in the field located at Brzostówka (BRZ) as sampled during the 3 years. The color represents the OTU intensity: Blue to red, min to max. The absence of OTU is displayed as white line. The intensity of the color represents the percentage of occurrence in the treatments. Samples were collected in **(A)** September 2014, **(B)** June 2015, **(C)** September 2015, **(D)** May 2016, and **(E)** July 2016.

In the samples collected in 2014 (after application), the number of bacteria OTUs present in ≥1 treatment samples was 22 in BRZ and 25 in NOW (Figures 1A, 2A). In total, 15 of them were common to both sites. The application of the two bioinocula induced a different response in terms of OTU intensity in the two sites: a general reduction of the intensity was observed in the samples from BRZ (for the 62 and 48% of the OTUs in the case of *B. brongniartii* and *B. bassiana*, respectively), while a general increase was observed in NOW (for the 72 and 48% of the OTUs in case of *B. brongniartii* and *B. bassiana*, respectively) (Table 1). Among the 15 OTUs common to both sites, only two of them responded similarly to the application of inocula in both sites (OTUs 71.7 and 144, with reduced intensity) while all others responded differently (either being enhanced or reduced after the application compared to the control) (Figures 1A, 2A and Table 1).

**TABLE 1:** Percentage of bacterial operational taxonomic units (OTUs) categorized according to the comparison of intensity with the control.

BRZ																														
	2014						2015												2016											
	T1						T1						T2						T1						T2					
	Unchanged	Reduced	Increased	Missing	Additional	Total	Unchanged	Reduced	Increased	Missing	Additional	Total	Unchanged	Reduced	Increased	Missing	Additional	Total	Unchanged	Reduced	Increased	Missing	Additional	Total	Unchanged	Reduced	Increased	Missing	Additional	Total
Br	33	62	5	0	10	21 (47)	23	31	23	8	15	13 (30)	0	64	18	18	0	11 (43)	39	0	54	0	8	13 (91)	17	17	67	0	0	6 (72)
Ba	38	48	5	10	10		8	62	15	0	15		27	46	18	9	0		23	15	31	23	8		17	0	67	17	0	
**NOW**																														
	**2014**						**2015**												**2016**											
	**T1**						**T1**						**T2**						**T1**						**T2**					
	**Unchanged**	**Reduced**	**Increased**	**Missing**	**Additional**	**Total**	**Unchanged**	**Reduced**	**Increased**	**Missing**	**Additional**	**Total**	**Unchanged**	**Reduced**	**Increased**	**Missing**	**Additional**	**Total**	**Unchanged**	**Reduced**	**Increased**	**Missing**	**Additional**	**Total**	**Unchanged**	**Reduced**	**Increased**	**Missing**	**Additional**	**Total**
Br	20	0	72	8	0	25 (57)	13	75	0	0	13	16 (48)	75	0	0	8	17	12 (42)	11	0	90	0	0	19 (98)	11	0	56	11	22	9 (90)
Ba	40	4	48	8	0		19	44	6	19	13		0	25	58	0	17		42	11	5	42	0		33	22	22	0	22	

The column “Total” indicates the total number of OTUs selected for the impact assessment according to the criteria specified in the text and, in brackets, the overall number of OTUs identified with terminal restriction fragment polymorphism (T-RFLP) analysis.

In 2015, a total of 16 and 12 OTUs were identified during the first and second sampling, respectively, in NOW, with nine of them common to both sampling times (Figures 1B, C). Both inocula induced a general reduction in the intensity of OTUs in the first sampling time: in 75% of OTUs in the case of *B. brongniartii* and 44% of OTUs in the case of *B. bassiana* (Table 1). The fall samples, different from the previous ones, showed no impact of *B. brongniartii* inoculation on OTU intensity, while *B. bassiana* induced an increase in the intensity of several OTUs compared to the control (Figure 1C and Table 1). Concerning the BRZ field, 13 and 11 OTUs were identified in the 2015 first and second sampling time, respectively, eight of which were common to both periods (Figures 2B, C). In the spring samples, the presence of *B. bassiana* induced a general reduction of the OTU intensity (on 62% of OTUs), while *B. brongniartii* resulted to induce a similar share among the three categories of changes (Table 1 and Figure 2B). In the fall samples, both inocula induced a reduction in the intensity in the majority of OTUs (64 and 46% for *B. brongniartii* and *B. bassiana*, respectively) with only few of them having unchanged or increased intensity (Figure 2C and Table 1).

In 2016, 19 OTUs were defined in May and 9 in July soil samples from NOW, three of which were common to both periods (Figures 1D, E). *B. brongniartii* induced an increased intensity in the majority of them in both sampling times, while *B. bassiana* affected the OTU intensity only to a limited extent, having the different categories of the changes a quite similar share in both sampling periods (Table 1 and Figures 1D, E). The samples from BRZ were characterized by 13 and 6 OTUs, respectively, for the first and second sampling times, none of them common to both periods (Figures 2D, E). The presence of *B. brongniartii* induced an increase of more than half or 67% of OTUs in the first and second sampling times, respectively. *B. bassiana*, instead, induced a relevant increase of OTU intensity only in the second sampling period, while not affecting them during the first sampling (Table 1 and Figures 2D, E).

Analyzing the impact across the seasons, the bioinocula resulted to induce a change in the intensity of the OTUs only few months after the application (i.e., in the T2 samples of 2015 and 2016) (Table 1). This tended to follow an increasing trend across the seasons, particularly in terms of the number of OTUs affected in the last sampling period. However, very few OTUs were consistently observed across the seasons in each field for at least three sampling periods (Table 1). Among them, five were common to both sites (OTUs 71.2, 105.4, 108.6, 139.1, and 175). However, their intensity in relation to the application of the two bioinocula was not consistently affected by them. In general, few OTUs were also not found or additionally present in the treated plots compared to the control in both sites during the different sampling periods.

### 3.2. Impact of inoculated species on soil fungal community

**FIGURE 3:**
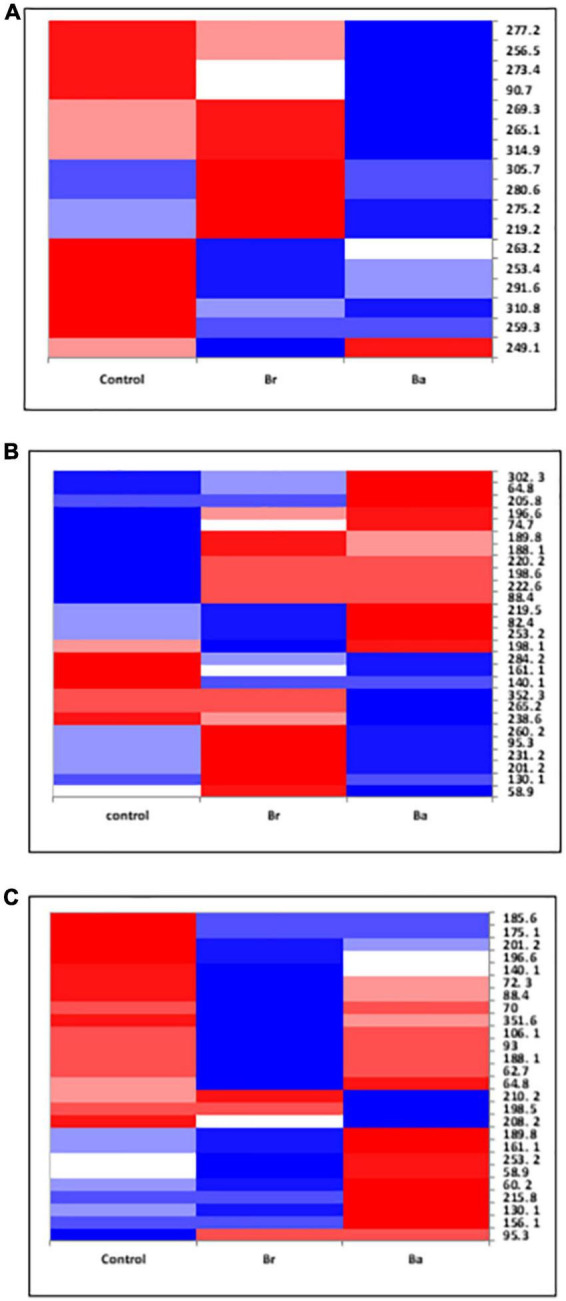
Heatmap displaying the occurrences of the common fungal operational taxonomic units (OTUs) identified, as T-RFs, in the soils treated with *Beauveria brongniartii* (Br) and *Beauveria bassiana* (Ba) bioinocula and untreated (Control) in the field located at Nowa Wola (NOW) as sampled during the 3 years. The color represents the OTU intensity: Blue to red, min to max. The absence of OTU is displayed as white line. The intensity of the color represents the percentage of occurrence in the treatments. Samples were collected in **(A)** September 2014, **(B)** June 2015, and **(C)** September 2015.

**FIGURE 4:**
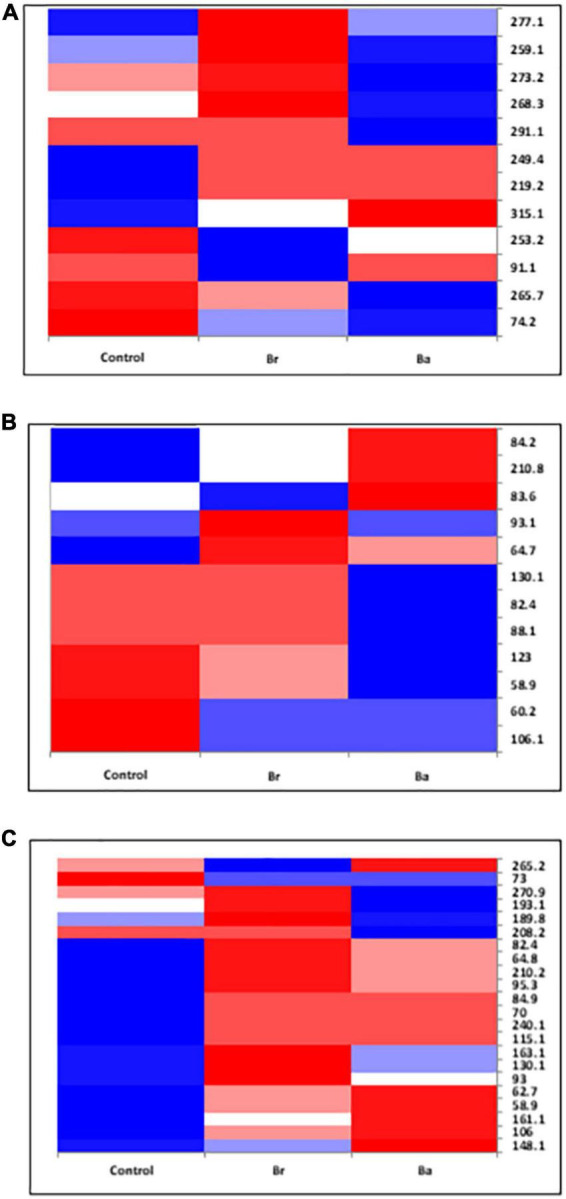
Heatmap displaying the occurrences of the common fungal operational taxonomic units (OTUs) identified, as T-RFs, in the soils treated with *Beauveria brongniartii* (Br) and *Beauveria bassiana* (Ba) bioinocula and untreated (Control) in the field located at Brzostówka (BRZ) as sampled during the 3 years. The color represents the OTU intensity: Blue to red, min to max. The absence of OTU is displayed as white line. The intensity of the color represents the percentage of occurrence in the treatments. Samples were collected in **(A)** September 2014, **(B)** June 2015, and **(C)** September 2015.

The samples collected in 2014 resulted to have 12 OTUs in BRZ and 17 in NOW (Figures 3A, 4A). In total, seven OTUs were common to both sites, and four of them responded similarly in terms of intensity to the application of the two inocula in both sites compared to the control. Considering the impact of each inoculum in comparison with the control, at both sites, the application of *B. brongniartii* induced limited changes, with a similar share among the three possible modifications (Table 2). On the other hand, the application of *B. bassiana* induced a higher intensity on the majority of OTUs in NOW, while in BRZ, the impact was on a lower number of OTUs, balanced among unchanged and changed (either increased or decreased).

**TABLE 2:** Percentage of fungal operational taxonomic units (OTUs) categorized according to the comparison of intensity with the control.

BRZ																		
	2014						2015											
	T1						T1						T2					
	Unchanged	Reduced	Increased	Missing	Additional	Total	Unchanged	Reduced	Increased	Missing	Additional	Total	Unchanged	Reduced	Increased	Missing	Additional	Total
Br	25	33	25	8	8	12 (50)	42	17	17	17	8	12 (203)	9	73	9	5	5	22 (266)
Ba	25	25	33	8	8		8	25	58	0	8		14	59	18	5	5	
**NOW**																		
	**2014**						**2015**											
	**T1**						**T1**						**T2**					
	**Unchanged**	**Reduced**	**Increased**	**Missing**	**Additional**	**Total**	**Unchanged**	**Reduced**	**Increased**	**Missing**	**Additional**	**Total**	**Unchanged**	**Reduced**	**Increased**	**Missing**	**Additional**	**Total**
Br	29	24	35	12	6	17 (60)	33	30	26	7	4	27 (265)	31	4	54	8	4	26 (405)
Ba	29	0	65	6	0		22	52	22	0	4		35	27	23	8	8	

The column “Total” indicates the total number of OTUs selected for the impact assessment according to the criteria specified in the text and, in brackets, the overall number of OTUs identified with terminal restriction fragment polymorphism (T-RFLP) analysis.

In 2015, the samples collected from the NOW site contained 27 and 26 OTUs in the first and second sampling times, respectively (Figures 3B, C), while those from the BRZ site resulted to contain 12 and 22 OTUs (Figures 4B, C). In NOW, twelve OTUs were common in both sampling times, while eight of them were common in the case of BRZ. However, though in BRZ, three of these common OTUs resulted to respond similarly to the application of both inocula in terms of intensity changes compared to the control in both sampling periods, in NOW, any OTU resulted to modify consistently the intensity for the sampling periods concerned (Figures 3, 4). The impact of the inocula on the intensity of the OTUs was different depending on the site and sampling period (Table 2). In NOW, *B. bassiana* induced a reduction to more than half OTUs in the first sampling, while in the second, the modifications were equivalent in share. An opposite pattern was instead induced by *B. brongniartii*, with a common percentage of the different modifications during the first sampling and an enhancement of the increased intensity of OTUs in the second sampling period. In BRZ, *B. bassiana* induced a diverging impact on the OTU intensity when comparing the first (higher share of increased intensity–58%) and second (higher share of decreased intensity–59%) sampling periods (Table 2). Considering *B. brongniartii*, its application did not modify the intensity for the majority of OTUs compared to the control (42%) during the first sampling period, while inducing a decrease in the intensity of the majority of OTUs (59%) in the second sampling period (Table 2). In 2016, we were unable to observe fungi OTUs in the samples collected by applying the criterium of finding at least one T-RF per sample and avoiding rare T-RFs. Only three OTUs were consistently detected across the sampling periods and only in the NOW field: 265.1, 219.2, and 253.4 (Figure 3). However, the impact of the two bioinocula on their intensity was inconsistent.

## 4. Discussion

### 4.1. Impact of entomopathogenic fungi inoculants on soil microbial communities

In applying the T-RFLP approach to soil inoculated with *B. bassiana* and *B. brongniartii*, we considered it useful for the analysis when at least one T-RF per sample was observed, indicating the presence of the target gene in the investigated samples, while we did not include rare T-RFs occurring in few specific samples (data not shown). This approach resulted in the lack of fungal OTUs from the samples collected in 2016. However, it is believed that the stringent criterium adopted in the study reduced the risk of a biased analysis and evaluation of the bioinocula impact on the microbial communities, which was possible for the other sampling periods and always for the bacteria community.

The presence of discriminant bands (OTU) could be associated with a putative species that may be characteristic of a certain soil sample. In a previous study (Tartanus et al., 2021), the digestion of the amplicons obtained from the soils treated with *B. bassiana* and *B. brongniartii* inocula resulted in different numbers of T-RFs for bacteria and fungi, ranging from 6 to 80 T-RFs, pointing to a wide genetic diversity of bacteria and fungi in these soils. Overall, the applied strains affected, to a different extent, the soil bacterial and fungal communities of the two investigated sites, which are characterized by different soil physical-chemical conditions, highlighting that the inoculative effect may depend also on the resident microbial community encountered by the inoculant and, consequently, by the interaction between it and the native community (Malusà et al., 2021). We have thus further analyzed the OTU data in an effort of identifying the possible effects of the bioinocula on the soil autochthonous microbiome with an approach that could be useful also to support other methods (e.g., NGS) to define the impact of bioinocula on the environment (Deising et al., 2017; Mawarda et al., 2020). Indeed, studies assessing the effects of applied microorganisms, particularly entomopathogenic fungi, on soil microbial communities have revealed only small or transient effects (Trabelsi and Mhamdi, 2013; Kröber et al., 2014; Zimmermann et al., 2016). Nevertheless, Mawarda et al. (2020) reported that 30% of studies using fingerprinting-based methods (e.g., TRFLP, DGGE, and TGGE) did not show any consistent effect of inoculation, pointing to a likely methodological limitation. In the case of EPF, the potential effects on the soil microbiome after the application were found to vary (Hu and St. Leger, 2002; Schwarzenbach et al., 2009; Hirsch et al., 2013; Mayerhofer et al., 2017, 2019). The results of this study point to similar conclusions, as the modifications observed in the OTU number and intensity were generally of limited effect, not consistent in the two studied fields and across the seasons considered. However, it is noteworthy to consider these results also in relation to the timing of bioinocula application and soil sampling and the soil characteristics of the fields, which are factors that could impact the potential effect of the bioinocula as well as their interactions with the native microbiome.

### 4.2. Impact of entomopathogenic fungi inoculants on soil bacteria community

The application of the bioinocula modified to a low extent the composition of bacterial communities after each application, as only few new or missing OTUs (about 1/10 of the OTUs identified) were detected in the inoculated soil (i.e., were triggered or not in inoculated soil) compared to the control across the whole sampling period. However, even though probably influenced by other factors, the inoculation affected the intensity of the bacterial OTUs, i.e., their quantitative contribution to the total bacteria community. The impact of both bioinocula in this respect could not be considered fully consistent across the seasons (sampling periods) and fields. However, considering the time passed since the application, some common patterns were highlighted in each field and sometimes also among them, showing that a certain impact could be attributed to the bioinocula. In 2016, the intensity was increased in the majority of the OTUs by both bioinocula in NOW, but also a significant percentage was not changed. The same amplitude of changes was observed in BRZ, only with the majority of OTUs having a decreased intensity. In the first sampling period of 2015, before the second application of the bioinocula, both sites showed a majority of OTUs with decreased intensity compared to the control and a similar number of unchanged OTU intensity. The impact of both bioinocula on bacteria OTU intensity for the BRZ samples was similar at the end of the 2015 season, 8 weeks after their application, but not for the NOW site, where the two bioinocula affected differently the bacterial community. In 2016, a similar impact on OTU intensity was again observed in both sites and both sampling periods in the plots treated with *B. brongniartii*. This was consistently observed also in *B. bassiana* plots, with the exception of the first sampling in NOW, when, however, an uncommon number of missing OTUs (i.e., not possible to determine considering the identification criteria) was found.

Draganova et al. (2008) reported that strains of *B. bassiana* showed varying degrees of impact on the intensity of significant groups of soil microorganisms (bacteria, actinomycetes, and fungi). Some strains (e.g., *B. bassiana* 224Re) showed no impact; in contrast, the strain *B. bassiana* 412 showed the most substantial stimulation effect on heterotrophic microorganisms, mineral nitrogen utilizing bacteria, free-living nitrogen-fixing microorganisms, and soil fungi, with 14, 15, 7, and 30 times higher density of these microorganisms compared to the control treatments, respectively. However, the same strain caused suppression of the cellulose degrading microorganisms compared to the non-inoculated soil. Isolates of *B. bassiana* had no or little influence on the microbial community composition and function in the rhizosphere up to 30 days after inoculation, as assessed by DGGE and microrespiration analyses (McKinnon et al., 2018). Moreover, the density dynamics of *B. bassiana* introduced into forest soil did not affect the densities of bacteria and actinomycetes (Shimazu et al., 2002). Considering that a significant decline in all detectable *Beauveria* strains was observed in the fields of the study using a PCR-based method (Tartanus et al., 2021), this may indicate that the inocula did not have a lasting effect. Such a hypothesis would be confirmed by the generally consistent similarities in OTU intensity among bioinocula and sites found on the samples collected in spring 2015 and 2016, before the new application, as well as by considering also only the OTUs common to different sampling periods and sites.

Entomopathogenic fungi from the genus *Beauveria* are considered common rhizosphere colonizers in many ecosystems (Jaronski, 2007; Zimmermann, 2007). They are generalist pathogens to various insect species, the cadavers of which may provide a source of nitrogen to plant roots through the fungal mycelial network from an insect cadaver (Barelli et al., 2016), as it has been shown for another entomopathogenic fungal genus–*Metarhizium* (Bruck, 2010; Behie et al., 2012). In this instance, the soil inoculated with EPF could provide more nutrients derived from their metabolism that modify the composition of the bacteria community (González-Guzmán et al., 2020). However, there is limited knowledge of interactions between EPF and other soil microorganisms (Lozano-Tovar et al., 2017). Another mechanism triggered by the EPF affecting the bacterial community could result from the modification of root exudates (composition or quantity). Indeed, the endophytic behavior of *B. bassiana* and its capacity to develop on root exudates, particularly in the absence of the insect host, would likely modify the soil chemical environment, leading to changes in the bacterial community (Vega, 2018; Rolfe et al., 2019).

The nutrient promotion effect may vary under different environmental conditions (Hossain et al., 2017; Yadav, 2020). Therefore, the different impacts observed in the various sampling periods and/or fields could be the expression of the interaction between the EPF activity and the environmental conditions. In some cases (e.g., during the 2015 season), the lower number of common OTUs (T-RFs) and their lowered intensity compared to the control or the previous sampling period could point to a higher genetic diversity promoted by the bioinocula or to the return of the microbial community to a pre-application equilibrium due to bioinoculum level decrease (Scheepmaker and Butt, 2010).

### 4.3. Impact of entomopathogenic fungi inoculants on soil fungal community

The impact of the two bioinocula on the fungal OTUs was quite limited, as only in BRZ 2015 the samples after the application showed a higher OTU number compared to the sample collected before the application. No pattern of influence emerged in analyzing the effect on OTU intensity. This situation was well highlighted during the last season (2016) when any specific OTU specifically influenced by the bioinocula was detected.

Several studies have detected limited or only transient effects on soil fungi after the application of bioinocula of both *B. bassiana* and *B. brongniartii* (Rai and Singh, 2002; Schwarzenbach et al., 2009; Hirsch et al., 2013). A *Metarhizium brunneum* (Hypocreales: Clavicipitaceae) strain formulated as fungus colonized barley kernels (FCBKs) used in a pot experiment showed to induce some changes in the fungal community structures (Mayerhofer et al., 2017), differently to the field application of the same formulation or previous reports indicating a lack of impact from the application of *Metarhizium anisopliae* sensu lato (Kirchmair et al., 2008). However, since the non-formulated fungal spores resulted in not affecting the fungal communities, it was suggested that the effects could mainly derive from the formulation carrier (i.e., the kernels). This could also have occurred in the present study, particularly in the *B. brongniartii* strain, which was also formulated as an FCBK. Moreover, time-related effects on the fungal and prokaryotic soil communities in the pot experiment were similar to or greater than the treatment effects (Mayerhofer et al., 2017). The following study confirmed this hypothesis, showing that neither changes in soil fungal or prokaryotic community structures nor relative sequence abundance of individual OTUs could be detected upon the application of *M. brunneum* formulated as FCBK (Mayerhofer et al., 2019). Seasonal changes in soil microbial composition in relation to the plant developmental stage were also found to exceed the effects of applied fungi and bacteria in bulk soil (Savazzini et al., 2009) as well as in the rhizosphere (Van Dillewijn et al., 2002; Grosch et al., 2006).

Microbial control usually implies the application of large amounts of infective propagules of a biological control agent to soils under treatment. For instance, about 10^12^–10^14^ propagules of EPF are applied per hectare, translating into 10^5^ spores per cm^2^ of soil (Jaronski, 2010). Such high loads of propagules may have unintended side effects, leading to changes in soil microbial community structures. The densities of total fungi were significantly increased by *B. bassiana* application in forest soils (Shimazu et al., 2002). A possible effect of the impact of the bioinocula could be the competition with native arbuscular mycorrhizal fungi, which was detected in the case of *B. bassiana* in oak and maize roots (Zitlalpopoca-Hernandez et al., 2017; Matek et al., 2019). A positive outcome of this kind of impact could be the antagonism with soil-borne fungal pathogens (Jaber, 2018; Wiberth et al., 2019) or the synergistic activity with other mycoparasite species (Krauss et al., 2004). Nevertheless, since the host mortality is dose-dependent (Jaronski, 2007; Inglis et al., 2009), it could also be postulated that the impact on the microbiome (or non-target organisms) is transient, as it emerged also from the present study, and decreasing as the inoculum levels decrease.

## 5. Conclusion

With regard to the impact on the bacterial community, both *Beauveria* species were not fully consistently affecting their composition across the seasons and fields tested. Nevertheless, some common patterns were pointed out in each field and, sometimes, also among them when considering the time elapsed from the bioinoculum application. The impact was even more inconsistent when analyzing the fungal community. It is thus concluded that the analyses performed pointed to a transient and limited effect of both *Beauveria* species on the soil microbial community even though some changes in the structure dynamic and frequency of soil bacterial and fungal OTUs emerged.

## Data availability statement

The original contributions presented in this study are included in the article/Supplementary material, further inquiries can be directed to the corresponding authors.

## Author contributions

LC, EM, and MT: conceptualization and methodology. LC and MT: analysis and investigation. All authors have wrote, reviewed, read, and agreed to the published version of the manuscript.
