# Supplementary material for: The impact of Beauveria species bioinocula on the soil microbial community structure in organic strawberry plantations
Source: Front Microbiol. 2023 Jan 11;13:1073386. doi: 10.3389/fmicb.2022.1073386 (PMC9874679; doi:10.3389/fmicb.2022.1073386)
Supplement: Supplementary file 1 [file Data_Sheet_1.DOCX]

Supplementary Material

**Table S1.** Primers used for TRFLP analysis, with annealing temperatures and target regions.

| Target group | | Primers | Primer sequence | amplicon length (bp) | Annealing  temp (°C) | Reference | Target  regions |
| --- | --- | --- | --- | --- | --- | --- | --- |
| Bacteria | 63F  1087R | | 5’AGGCCTAACACATGCAAGTC3’  5’CTCGTTGCGGGACTTACCCC3’ | 1000 bp |  | Singh et al., 2006 | 16S rRNA |
| Fungi | ITS1  ITS4 | | 5’CTTGGTCATTTAGAGGAAGTAA3’  5’TCCTCCGCTTATTGATAT GC3’ | 700 bp | 55°C | Gardens and Brums 1993 | ITS1 |
